# Advancing Natural Bioactive Formulations: Innovations in Agri-Food-Pharma for Enhanced Health and Sustainability

**DOI:** 10.3390/bioengineering12040405

**Published:** 2025-04-11

**Authors:** Kandi Sridhar, Baskaran Stephen Inbaraj, Minaxi Sharma

**Affiliations:** 1 Department of Food Technology, Karpagam Academy of Higher Education (Deemed to be University), Coimbatore 641021, India; 2 Department of Food Science, Fu Jen Catholic University, New Taipei City 242062, Taiwan; 3 Research Centre for Life Science and Healthcare, Nottingham Ningbo China Beacons of Excellence Research and Innovation Institute (CBI), University of Nottingham Ningbo China, Ningbo 315000, China

Over the years, it has been confirmed that bioactive compounds play a pivotal role in human health, particularly in the prevention of chronic diseases. These bioactive compounds exhibit diverse biological activities and have found applications in multiple sectors, including nutraceuticals, biopharmaceuticals, biosurfactants, biostimulants, and cosmeceuticals. With the growing demand for natural and sustainable health solutions, agri-food-pharma-health industries are actively exploring natural bioactive compound sources, focusing on their identification, formulation, and development into bioactive-enriched agri-food-pharma supplements.

**The Growing Importance of Natural Bioactive Compounds**

The increasing recognition of bioactive compounds in health and nutrition has prompted extensive research into novel and bioactive natural sources. Progress in the identification and production of these bioactive compounds is crucial for enhancing their application in the agri-food-pharma-health industries. Observations from natural ecosystems and the diversity of bioactive complexes have inspired industries and researchers to engineer novel bioactives from microbial and plant sources. These efforts are further supported by technological advancements that facilitate efficient extraction, purification, and formulation strategies, thereby broadening the scope of bioactive applications. However, the diverse industrial uses of these bioactive compounds in agri-food-pharma further requires efficient formulation techniques to ensure stability, bioavailability, and targeted efficacy. Therefore, multidisciplinary research areas integrating biochemistry, molecular biology, and bioprocess engineering should aim to enhance bioactive compound applications. Hence, engineered approaches have been employed to improve the biological properties of bioactives. To provide a comprehensive perspective on bioactive formulations, this Special Issue on “Bioactive Formulations in Agri-Food-Pharma: Source and Applications, Volume III” aims to compile cutting-edge research on the production, formulation, and application of natural bioactive compounds from various bioresources. This Special Issue, set to be published in *Bioengineering* (ISSN 2306-5354) under the “Biochemical Engineering” Section, highlights the latest advancements in this area, including the following:

- Recent developments in the production of natural bioactives from various biological sources.
- Engineered strategies for enhancing the biological activity and functional properties of bioactive compounds.
- Novel industrial applications and bioprocessing tools for improved bioactive yields.
- Genomic- and molecular-level insights into the regulation of bioactive formulations.
- Innovative formulation approaches in optimizing bioactive stability, delivery, and efficacy.

This Special Issue has attracted contributions from leading researchers worldwide, demonstrating the significance of bioactive compounds in agri-food-pharma industries. A total of fourteen submissions were received, in which five papers (two review articles and three research papers) were accepted after rigorous peer review. These publications collectively provide significant insights into emerging trends, challenges, and future directions in bioactive formulation research. The published articles in this Special Issue are available as open access publications under the Creative Commons Attribution (CC BY) license with full unrestricted sharing, adaptation, and distribution. By making these contributions freely accessible, this initiative fosters a greater exchange of knowledge among researchers, industry professionals, and the public, strongly supporting global advancements of bioactive formulations. The key findings and implications of the research and review articles published in this Special Issue are summarized below.

A study by Wei et al. [1] investigated turmeric-derived exosome-like nanoparticles (TELNs) focusing on extraction and purification using centrifugation and ultracentrifugation, achieving an extraction rate of 1.71 mg/g of peeled turmeric. The study employed multi-omics approaches to analyze the physicochemical properties of TELNs, revealing their typical teacup-like morphology with an average particle size of 183.20 nm and a zeta potential of −17.60 mV. The lipid composition of TELNs was notably rich in key lipids including phosphatidylethanolamine (PE) and triglycerides (TGs), while the protein content was primarily composed of curcumin synthase and other proteins involved in secondary metabolite biosynthesis. Additionally, the study identified essential genes for curcumin biosynthesis, such as curcumin synthase (CURS) and diketocoenzyme A synthase (DCS). The study highlighted the presence of various small-molecule compounds (curcumin and its analogs), which contributed to the therapeutic potential of TELNs. Similarly, another study by Hwang [2] focused on the antibacterial properties of coumarin derivatives synthesized from lime peel and their effectiveness against various food-poisoning bacteria. The results showed that some coumarin derivatives exhibited significant antibacterial activity with the minimum inhibitory concentration (MIC) measured for specific derivatives against different bacterial strains. The study also emphasized the importance of understanding the structure–activity relationship (SAR) of coumarin derivatives to optimize their antibacterial activity. Overall, both studies provide important insights into the antibacterial properties of coumarin derivatives and their potential applications in food safety and preservation. Additionally, it highlighted the significance of TELNs as a source of bioactive compounds with potential applications in drug delivery and therapeutic development. By utilizing multi-omics approaches to characterize TELNs, the study marks a pioneering effort in understanding their functional properties and biomedical potential.

Another study explored the microencapsulation of extra virgin olive oil using maltodextrin (MD) and whey protein isolates (WPIs) as wall materials [3]. The key findings indicated that the stability, viscosity, and droplet size of the emulsions significantly influenced the characteristics of the resulting microcapsules. The highest encapsulation efficiency (EE) was achieved with a 50% MD and 50% WPI emulsion dehydrated by spray drying, while the moisture content of the microcapsules increased with higher MD proportions. The study also observed that microcapsules with a higher WPI content exhibited larger sizes and poorer flowability, while those with higher MD content showed reduced bulk and tapped densities. Morphological analysis revealed that microcapsules produced by spray drying were generally spherical with smooth surfaces, whereas those produced by freeze-drying were flat flakes with irregular surfaces. The study highlighted the potential of using MD and WPI combinations for the effective microencapsulation of olive oil, which can help maintain its quality and biological activity against oxidation, thus offering crucial insights for food processing and preservation applications.

Nakashio et al. [4] investigated the impact of additives (i.e., milk, sugar, and lemon juice) to black tea on its ability to inactivate SARS-CoV-2, particularly the Omicron variant. The study showed that while black tea significantly reduced the infectivity of the virus, the addition of milk negated this effect due to the binding of milk casein to galloylated theaflavins (gTFs), preventing them from inactivating the virus. The study concluded that consuming black tea without milk may be beneficial for individuals infected with SARS-CoV-2 as it could help inactivate the virus in saliva, highlighting the importance of dietary choices in viral transmission.

Garg et al. [5] reviewed the integration of nanotechnology in agriculture by focusing on nano-biofertilizers (NBFs), which combine nanoparticles with biofertilizers to enhance plant growth and resilience against stressors. The review highlighted advancements in the formulation, fabrication, and characterization of NBFs, emphasizing their mechanisms of action (i.e., nitrogen fixation and improved nutrient availability), which addressed challenges like soil degradation and the need for higher agricultural yields. The review concluded by outlining the growing interest in NBFs, with significant research activity in the field since 2020, and called for further studies to understand the interactions between NBFs and plant systems to optimize agricultural practices and contribute to food security.

In conclusion, the collective findings from this Special Issue demonstrated the significant role of natural compounds and innovative technologies in enhancing health and agricultural practices. The studies highlighted the importance of understanding the interactions between dietary components, the potential of nanotechnology in agriculture, and the antibacterial properties of plant-derived compounds. As the demand for sustainable and natural solutions continues to grow, further research should be conducted to optimize the applications of these findings. Areas of interest include, for example, exploring the SARs of natural compounds, assessing their safety and efficacy, and developing strategies for their integration into food systems and agricultural practices. Ultimately, these efforts could lead to improved health outcomes, enhanced food safety, and sustainable agricultural practices, contributing to a healthier and more secure future.
